# Correlation analysis of PGC-1β, HIF-1α and RETN with the degree of joint destruction in gouty arthritis

**DOI:** 10.5937/jomb0-60976

**Published:** 2026-04-15

**Authors:** Jian Bian, Jianguo Zhang, Bo Zhang, Hongfei Shi

**Affiliations:** 1 Department of Orthopedic Surgery, Nanjing Drum Tower Hospital Clinical College of Nanjing Medical University, Nanjing Jiangbei Hospital, China; 2 Department of Orthopedic Surgery, The First Affiliated Hospital of Zhengzhou University. No. 1, China

**Keywords:** PGC-1β, HIF-1α, RETN, joint destruction, gouty arthritis, correlation analysis, PGC-1β, HIF-1α, RETN, destrukcija zglobova, giht, analiza korelacije

## Abstract

**Background:**

To explore the expression levels of resistin (RETN), hypoxia-inducible factor-la (HIF -l<span style="color: rgb(32, 33, 34); font-family: sans-serif; font-size: 16px; background-color: rgb(248, 249, 250);">α</span> ), and peroxi-some proliferator-activated receptor g coactivator-1<span style="color: rgb(32, 33, 34); font-family: sans-serif; font-size: 16px; background-color: rgb(248, 249, 250);">β</span> (PGCip ) in gouty arthritis (GA) patients and to analyse their cor-relations with the degree of joint destruction.

**Methods:**

The GA group consisted of 134 patients with GA who were admitted to the hospital between January 2023 and October 2024, while the control group consisted of 134 healthy patients who underwent physical examination in the hospital over the same time period. Serum PGC-1<span style="color: rgb(32, 33, 34); font-family: sans-serif; font-size: 16px; background-color: rgb(248, 249, 250);">β</span>, HIF-1<span style="color: rgb(32, 33, 34); font-family: sans-serif; font-size: 16px; background-color: rgb(248, 249, 250);">α</span>, and RETN expression levels were compared. The expression levels of PGC-1<span style="color: rgb(32, 33, 34); font-family: sans-serif; font-size: 16px; background-color: rgb(248, 249, 250);">β</span>, HIF-1a and RETN in the serum and synovial fluid of patients with different clinical characteristics in the GA group were compared. The degree of joint destruction was categorised into 78 cases in the severe GA subgroup and 56 cases in the mild GA subgroup based on the VAS score of the chief complaint pain scale. Compared with PGC-1<span style="color: rgb(32, 33, 34); font-family: sans-serif; font-size: 16px; background-color: rgb(248, 249, 250);">β</span>, HIF-1<span style="color: rgb(32, 33, 34); font-family: sans-serif; font-size: 16px; background-color: rgb(248, 249, 250);">α</span>, RETN, and bone destruction factors [<span style="color: rgb(32, 33, 34); font-family: sans-serif; font-size: 16px; background-color: rgb(248, 249, 250);">β</span>-crosslinking degradation products (P-CTX), tartrate-resistant acid phosphatase-5b (TRACP5b), and nuclear factor p receptor activator ligand (RANKL)], and inflammatory factors with different degrees of joint destruction, the expression levels of 1p (IL-1 <span style="color: rgb(32, 33, 34); font-family: sans-serif; font-size: 16px; background-color: rgb(248, 249, 250);">β</span>) were analysed. The correlations between PGC-1<span style="color: rgb(32, 33, 34); font-family: sans-serif; font-size: 16px; background-color: rgb(248, 249, 250);">β</span>, HIF-1<span style="color: rgb(32, 33, 34); font-family: sans-serif; font-size: 16px; background-color: rgb(248, 249, 250);">α</span>, and RETN in serum and synovial fluid, as well as the degree of joint destruction, bone destruction factors, and inflamma-tory factors, were analysed.

**Results:**

The expression level of serum PGC-1<span style="color: rgb(32, 33, 34); font-family: sans-serif; font-size: 16px; background-color: rgb(248, 249, 250);">β</span> in the GA group was lower than that in the control group, while the expression levels of serum HIF-1<span style="color: rgb(32, 33, 34); font-family: sans-serif; font-size: 16px; background-color: rgb(248, 249, 250);">α</span> and RETN were greater than those in the control group (P&lt; 0.05). The expression levels of PGC-1<span style="color: rgb(32, 33, 34); font-family: sans-serif; font-size: 16px; background-color: rgb(248, 249, 250);">β</span> in the serum of GA patients at different clinical stages, affected joints, disease courses and annual attack frequencies. The expression levels of <span style="color: rgb(32, 33, 34); font-family: sans-serif; font-size: 16px; background-color: rgb(248, 249, 250);">β</span>-CTX and TRACP5b in the serum of GA patients at different clinical stages, affected joints, disease courses and annual attack frequencies, and in the severe GA subgroup were greater than those in the mild GA subgroup (P&lt; 0.05). RANKL expression was lower than in the mild GA subgroup (P&lt; 0.05). The serum and synovial fluid levels of PGC-1 <span style="color: rgb(32, 33, 34); font-family: sans-serif; font-size: 16px; background-color: rgb(248, 249, 250);">β</span>, P-CTX, TRACP5b, TNF-<span style="color: rgb(32, 33, 34); font-family: sans-serif; font-size: 16px; background-color: rgb(248, 249, 250);">α</span>, and IL-1<span style="color: rgb(32, 33, 34); font-family: sans-serif; font-size: 16px; background-color: rgb(248, 249, 250);">β</span> were negatively cor-related with the degree of joint destruction and positively cor-related with the level of RANKL. HIF-1<span style="color: rgb(32, 33, 34); font-family: sans-serif; font-size: 16px; background-color: rgb(248, 249, 250);">α</span> and RETN exhibited a negative correlation with RANKL and a positive correlation with the degree of joint degradation, as well as with <span style="color: rgb(32, 33, 34); font-family: sans-serif; font-size: 16px; background-color: rgb(248, 249, 250);">β</span>-CTX, TRACP5b, TNF-<span style="color: rgb(32, 33, 34); font-family: sans-serif; font-size: 16px; background-color: rgb(248, 249, 250);">α</span>, and IL-1<span style="color: rgb(32, 33, 34); font-family: sans-serif; font-size: 16px; background-color: rgb(248, 249, 250);">β</span>.

**Conclusions:**

PGC-1<span style="color: rgb(32, 33, 34); font-family: sans-serif; font-size: 16px; background-color: rgb(248, 249, 250);">β</span>, HIF-1<span style="color: rgb(32, 33, 34); font-family: sans-serif; font-size: 16px; background-color: rgb(248, 249, 250);">α</span> and RETN are abnormally expressed in patients with GA and are closely related to the degree of joint destruction, bone destruction factors and inflammatory factors. They are expected to become reli-able indicators for evaluating the occurrence and progres-sion of GA.

## Abstract (sr)

**Uvod:**

Cilj istraživanja bio je da se ispitaju nivoi ekspresije rezistina (RETN), hipoksijom inducibilnog faktora-1a (HIF-1<span style="color: rgb(32, 33, 34); font-family: sans-serif; font-size: 16px; background-color: rgb(248, 249, 250);">α</span>) i koaktivatora-1<span style="color: rgb(32, 33, 34); font-family: sans-serif; font-size: 16px; background-color: rgb(248, 249, 250);">β</span> receptora g aktiviranog proliferatorom peroksizoma (PGC-1<span style="color: rgb(32, 33, 34); font-family: sans-serif; font-size: 16px; background-color: rgb(248, 249, 250);">β</span>) kod pacijenata sa gihtom, kao i da se utvrdi njihova povezanost sa stepenom destrukcije zglobova.

**Metode:**

Istraživanje je obuhvatilo 134 pacijenta sa dijagnostikovanim gihtom, hospitalizovana u periodu od januara do oktobra 2024. godine (grupa sa gihtom), i 134 zdrava ispitanika koji su u istom periodu obavili sistematski pregled (kontrolna grupa). Upoređivani su nivoi ekspresije PGC-1<span style="color: rgb(32, 33, 34); font-family: sans-serif; font-size: 16px; background-color: rgb(248, 249, 250);">β</span>, HIF-1<span style="color: rgb(32, 33, 34); font-family: sans-serif; font-size: 16px; background-color: rgb(248, 249, 250);">α</span> i RETN u serumu između posmatranih grupa. Dodatno su analizirani nivoi navedenih biomarkera u serumu i sinovijalnoj tečnosti pacijenata sa različitim kliničkim karakteristikama unutar grupe sa gihtom. Na osnovu vrednosti na VAS skali bola, pacijenti su svrstani u podgrupu sa teškim oblikom gihta (n=78) i podgrupu sa blagim oblikom gihta (n = 56). Poređivani su nivoi ekspresije PGC-1<span style="color: rgb(32, 33, 34); font-family: sans-serif; font-size: 16px; background-color: rgb(248, 249, 250);">β</span>, HIF-1<span style="color: rgb(32, 33, 34); font-family: sans-serif; font-size: 16px; background-color: rgb(248, 249, 250);">α</span>, RETN, markera koštane destrukcije [p-ukrsteni proizvodi degradacije kolagena (<span style="color: rgb(32, 33, 34); font-family: sans-serif; font-size: 16px; background-color: rgb(248, 249, 250);">β</span>-CTX), tartrat-rezistentna kiselinska fosfataza-5b (TRACP5b) i ligand receptora aktivatora nuklearnog faktora <span style="color: rgb(32, 33, 34); font-family: sans-serif; font-size: 16px; background-color: rgb(248, 249, 250);">β</span> (RANKL)], kao i inflamatornih citokina, uključujući interleukin-1p (IL-1 <span style="color: rgb(32, 33, 34); font-family: sans-serif; font-size: 16px; background-color: rgb(248, 249, 250);">β</span>). Analizirane su korelacije između nivoa PGC-1<span style="color: rgb(32, 33, 34); font-family: sans-serif; font-size: 16px; background-color: rgb(248, 249, 250);">β</span>, HIF-1<span style="color: rgb(32, 33, 34); font-family: sans-serif; font-size: 16px; background-color: rgb(248, 249, 250);">α</span> i RETN u serumu i sinovijalnoj tečnosti, stepena oštećenja zglobova, markera koštane destrukcije i inflamatornih faktora.

**Rezultati:**

Nivo ekspresije PGC-1P u serumu pacijenata sa gihtom bio je značajno niži u poređenju sa kontrolnom grupom, dok su nivoi HIF-1<span style="color: rgb(32, 33, 34); font-family: sans-serif; font-size: 16px; background-color: rgb(248, 249, 250);">α</span> i RETN bili značajno visi (P &lt; 0 ,0 5 ). Ekspresija PGC-1<span style="color: rgb(32, 33, 34); font-family: sans-serif; font-size: 16px; background-color: rgb(248, 249, 250);">β</span> pokazala je statistički značajne razlike u zavisnosti od kliničkog stadijuma bolesti, broja zahvacenih zglobova, dužine trajanja bolesti i godisnje učestalosti napada. Vrednosti P-CTX i TRACP5b u serumu bile su vise u podgrupi sa teskim oblikom gihta nego u podgrupi sa blagim oblikom (P&lt; 0,05), dok je ekspresija RANKL bila niža (P &lt; 0,05). Nivoi PGC-1<span style="color: rgb(32, 33, 34); font-family: sans-serif; font-size: 16px; background-color: rgb(248, 249, 250);">β</span>, <span style="color: rgb(32, 33, 34); font-family: sans-serif; font-size: 16px; background-color: rgb(248, 249, 250);">β</span>-CTX, TRACP5b, TNF-<span style="color: rgb(32, 33, 34); font-family: sans-serif; font-size: 16px; background-color: rgb(248, 249, 250);">α</span> i IL-1<span style="color: rgb(32, 33, 34); font-family: sans-serif; font-size: 16px; background-color: rgb(248, 249, 250);">β</span> u serumu i sinovijalnoj tečnosti pokazali su negativnu korelaciju sa stepenom destrukcije zglobova, ali pozitivnu korelaciju sa nivoom RANKL. Suprotno tome, HIF-1<span style="color: rgb(32, 33, 34); font-family: sans-serif; font-size: 16px; background-color: rgb(248, 249, 250);">α</span> i RETN su bili negativno korelisani sa RANKL, a pozitivno sa stepenom destrukcije zglobova i nivoima P-CTX, TRACP5b, TNF-<span style="color: rgb(32, 33, 34); font-family: sans-serif; font-size: 16px; background-color: rgb(248, 249, 250);">α</span> i IL-1<span style="color: rgb(32, 33, 34); font-family: sans-serif; font-size: 16px; background-color: rgb(248, 249, 250);">β</span>.

**Zaključak:**

Biomarkeri PGC-1<span style="color: rgb(32, 33, 34); font-family: sans-serif; font-size: 16px; background-color: rgb(248, 249, 250);">β</span>, HIF-1<span style="color: rgb(32, 33, 34); font-family: sans-serif; font-size: 16px; background-color: rgb(248, 249, 250);">α</span> i RETN pokazuju značajnu disbalansiranu ekspresiju kod pacijenata sa gihtom i u tesnoj su korelaciji sa stepenom destrukcije zglobova, markerima kostane resorpcije i inflamatornim faktorima. Ovi biomarkeri mogu predstavljati potencijalne bioloske pokazatelje od dijagnostičkog i prognostičkog značaja za procenu nastanka i progresije gihta.

## Introduction

Gouty arthritis (GA) is highly prevalent in individuals approximately 50 years old and is characterised by recurrent attacks, with a tendency to affect younger people [1]. Due to the continuous increase in blood uric acid levels, it is deposited in joints, subcutaneous soft tissues, and other areas, constantly aggravating joint pain and bone destruction, and subsequently affecting patients' daily activities [2]. The pathogenesis of GA is complex. According to studies, local hypoxia and inflammatory reactions are directly linked to the development and course of GA [3]. The coactivator transcription factor peroxisome proliferator-activated receptor γ coactivator-1β (PGC-1β) is involved in the regulation of energy metabolism and cytokine signal transduction [4]. Hypoxia-inducible factor-1α (HIF-1α) is a nuclear transcription factor that is produced under hypoxic conditions and can mediate the response of cells to hypoxia [5][6]. Resistin (RETN) plays a role in regulating inflammatory responses in inflammatory diseases [7][8][9].

The associations between PGC-1β, HIF-1α, and RETN and the extent of joint degeneration in GA have not been extensively studied in clinical trials to date. This study investigated the expression of PGC-1β, HIF-1α, and RETN in GA and their relationship with the degree of joint destruction.

## Materials and methods

### General information

The GA group consisted of 134 GA patients who were admitted to our institution between January 2023 and October 2024. Comprising 53 females and 81 males, ages 43-68, with an average age of 23.16±1.13 kg/m^2^ and a body mass index of 19-26 kg/m^2^. Another 134 healthy individuals who underwent health check-ups at our hospital, including 74 males and 60 females, aged 45-68 years, with an average age of 56.19±5.25 years and a body mass index of 21-26 kg/m^2^, with an average of 23.28±0.95 kg/m^2^. The uric acid in the GA group ranged from 410 to 590 μmol/L, with an average of 493.84±41.60 μmol/L. Disease course: Fifty-three patients (39.55%) had a disease course of 5 years, and 81 patients (60.45%) had a disease course of <5 years. Affected joints: 54 patients (40.30%) with ≥5 joints and 80 patients (59.70%) with <5 joints. The annual incidence frequency was as follows: 62 patients (46.27%) had ≥3 cases, and 72 patients (53.73%) had <3 cases. Clinical stage: Eighty-three patients in the acute stage (61.94%) were in the chronic phase, 33 patients (24.63%) were in the acute stage, and 18 patients (13.43%) were in the early stage.

Inclusion criteria: (1) Consistent with a GA diagnosis; (2) No use of hormone drugs in the past month; (3) Age >18 years.

Exclusion criteria: (1) Had a history of joint surgery; (2) Had accompanying malignant tumors; (3) Had other types of arthritis, such as osteoarthritis or rheumatoid arthritis; (4) Had cerebrovascular diseases and abnormal functions of the liver, heart, kidneys, etc.; (5) Had secondary gout caused by cardiovascular diseases or kidney diseases; (6) Unable to tolerate the examination procedures of this study; (7) Had mental abnormalities.

This study complies with the requirements of the Declaration of Helsinki and was signed and approved by the hospital ethics committee [2022-745-15], participants and their families.

### Sample collection

The supernatant was frozen and stored for testing. Synovial fluid collection: For patients in the GA group, synovial fluid collection was performed. They were placed in a supine position. Under aseptic conditions, the painful joint was punctured to collect 1 mL of light yellow synovial fluid, which was frozen and stored for future use.

### Detection of PGC-1β, HIF-1α and RETN

The expression levels of PGC-1β, HIF-1α and RETN in the serum and synovial fluid of the subjects were determined via an enzyme-linked immunosorbent assay (ELISA) kit. The RT-96A microplate reader from Shenzhen Mindray was used, and the kit was purchased from BGI Technology Co., Ltd. in Shanghai.

After obtaining the synovial tissue from patients with gouty arthritis during the operation, it was immediately fixed in 4% paraformaldehyde (Sigma-Aldrich, P6148) for 24 hours, dehydrated in a gradient of ethanol (Sinopharm Group, 10009218/10009208), and embedded in paraffin (Leica, 39601006). After collecting 5 mL of fasting venous blood, let it stand at 4 for 30 minutes and then centrifuge at 3000xg for 15 minutes (centrifuge:) (Eppendorf 5424R), take the supernatant, aliquot into pyrogen-free EP tubes (Corning, 430790), and freeze at -80°C (Thermo Scientific ULT 1490) for testing.

### Immunohistochemical detection (PGC-1β, HIF-1α)

Continuous sections of paraffin blocks (5 μm thickness, Leica RM2235) were adhered to polylysine slides (Servicebio, G6010). Citrate buffer (10 mM, pH 6.0, Beyotime, P0083) 95°C water bath for 20 minutes. Block with 3% BSA (Sigma-Aldrich, A8020)/PBS for 30 minutes. Anti-pgc-1β rabbit monoclonal antibody (Abcam, ab176328, 1:200 dilution), anti-HIF-1α mouse monoclonal antibody (CST, 14179, 1:150 dilution), incubated overnight at 4°C in a wet box (16 hours). HRP-labelled sheep anti-rabbit/mouse IgG (ZSBG-BIO, PV-6001/PV-6002), incubated at 37°C for 1 hour. DAS Colour development kit (Vector Laboratories, SK-4105), controlled colour development time under microscope (Olympus BX53). Image-Pro Plus 6.0 software analysed the average optical density (IOD/Area).

### Serum RETN detection (ELISA method)

Human Resistin (RETN) ELISA Kit (R&D Systems, DRSN00). Standard gradient dilution (0-20 ng/mL), serum sample 100 pL/ well, multi-well detection, biotinylated antibody incubation (37°C, 2 hours), Streptavidin-HRP (R&D, DY998) reaction (37°C, 20 minutes). The TMB substrate (R&D, DY999) developed colour for 10 minutes, and the reaction was terminated with 2N H_2_SO_4_. The OD value was determined by a 450 nm wavelength microplate reader (BioTek Synergy H1), and the concentration was calculated by fitting the standard curve with four parameters.

### Detection of bone destruction factors and inflammatory factors

Five millilitres of fast elbow venous blood were collected, and the serum was separated by centrifugation. Bone destruction factors [β-crosslinking degradation products (β-CTX), tartrate-resistant acid phosphatase-5b (TRACP5b), and nuclear factor kB receptor-activating factor ligand (RANKL)C and inflammatory factors [tumour necrosis factor-α (TNF-α) and interleukin-1β (I)] were detected via ELISA 1-1β)].

Type I collagen carboxyl-terminal peptide (CTX-I): Serum ELISA (CUSABIO, CSB-E13130h), tartrate-resistant acid phosphatase 5b (TRACP5b): Plasma ELISA (MyBioSource, MBS2511526), IL-1β, TNF-α, IL-6: Serum multiplex liquid microarray (R&D Systems, LXSAHM-06).

Serum/plasma separation: EDTA anticoagulant tube (BD, 367856) for blood collection, centrifuge at 3000xg at 4°C for 15 minutes (Thermo ST 16R). Sample/standard 25 μL + microsphere mixture 25 μL, incubate at room temperature with shaking (500 rpm) for 2 hours, PE-labelled detection antibody (37°C, 1 hour).

### Biochemical indicators

1. Comparison of serum biomarkers: Serum levels of PGC-1β, HIF-1α, and RETN were compared between the two groups.
2. Analysis by clinical characteristics: The expression levels of PGC-1β, HIF-1α, and RETN in both serum and synovial fluid were compared among patients in the gouty arthritis (GA) group with different clinical characteristics.
3. Assessment by severity of joint destruction: Based on the visual analogue scale (VAS) score for pain, patients in the GA group were divided into two subgroups: 78 cases in the severe GA subgroup and 56 cases in the mild GA subgroup. The expression levels of PGC-1β, HIF-1α, RETN, bone destruction markers, and inflammatory factors were compared across these subgroups to evaluate the association with the degree of joint damage.
4. Correlation analysis: The relationships between serum and synovial fluid levels of PGC-1β, HIF-1α, and RETN and the degree of joint destruction, bone resorption markers, and inflammatory factors were further analysed.

### Measurement methods

- Serum uric acid (UA): Uricase-POD method (Roche Diagnostics, Cobas c702 reagent, item No. 04460785)
- C-reactive protein (CRP): Latex-enhanced immunoturbidimetric method (Siemens, ADVIA 2400 system reagent, item No. 11030132)
- Erythrocyte sedimentation rate (ESR): Wei's method (vacuum blood sedimentation tube, Greiner bio-One, item No. 455076)
- Liver and kidney function:<br>° Alanine aminotransferase (ALT) / Aspartate aminotransferase (AST): IFCC rate method (Roche, item Nos. 20764973 / 20764986)<br>° Creatinine (Cr): Enzymatic method(Roche, item No. 11775685)

### Statistical analysis

The χ^2^ test was used to analyse count data, which are typically expressed as percentages or the number of cases. The Spearman and Pearson correlation was used to analyse the relationships between serum, synovial fluid PGC-1β, HIF-1α, and RETN levels and the degree of joint destruction, bone destruction factors, and inflammatory factors. Bilateral tests were used, with a test level of α = 0.05.

## Results

### Comparison of the expression levels of PGC-1β, HIF-1α and RETN in the serum between the two groups

The expression level of serum PGC-1β in the GA group was lower than that in the control group, while the levels of RETN and HIF-1α were greater than those in the control group (P<0.05) - see Table 1.

**Table 1:** Comparison of expression levels of serum PGC-1β, HIF-1α and RETN between the two groups (x̄ ± s).

Group	n	PGC-1β (pg/mL)	RET (ng/mL)	HIF-1α (ng/L)
Group GA	134	2.02±0.64	25.64±6.08	43.55±9.58
Control group	134	3.28±0.81	13.23±3.10	26.68±8.44
t	-	14.129	21.049	15.295
P	-	≤0.001	<0.001	≤0.001

### Comparison of serum PGC-1β, HIF-1α and RETN expression levels in GA patients with different clinical characteristics

Comparisons of the expression levels of serum PGC-1β, HIF-1α, and RETN in GA patients with different clinical stages revealed statistically significant variations (P<0.05) in illness courses and annual attack frequencies - see Table 2.

**Table 2:** Comparison of serum PGC-1β, HIF-1α and RETN expression levels in patients with different clinical characteristics in the GA group (x̄ ± s).

Clinical features	n	PGC-1β (pg/mL)			HIF-1α (ng/L)			RETN (ng/mL)		
		x̄±s	t/F	P	x̄±s	t/F	P	x̄±s	t/F	P
Clinical staging			20.342	0.001		72.806	0.001		47.050	0.001
Acute stage	83	1.85±0.53			45.38±3.18			27.64±2.87		
Chronic phase	33	2.04±0.59			43.42±3.60			23.62±4.66		
Intermission period	18	2.76±0.56			35.35±2.37			20.02±2.04		
Affected joints (number)			2.602	0.010		5.693	0.001		9.371	0.001
≥5	54	1.86±0.54			45.06±2.71			27.34±1.51		
<5	80	2.13±0.62			42.53±2.39			24.52±1.83		
Course of the disease<br>(years)			3.323	0.001		4.370	0.001		6.982	<0.001
≥5	53	1.82±0.51			44.92±2.83			27.14±1.89		
<5	81	2.16±0.62			42.65±3.01			24.71±2.02		
Annual frequency of<br>attacks (times)			4.571	0.001		5.256	<0.001		9.563	≤0.001
≥3	62	1.77±0.52			44.85±2.57			27.02±1.37		
<3	72	2.24±0.65			42.43±2.73			24.49±1.65		

### Comparison of the expression levels of PGC-1β, HIF-1α and RETN in the synovial fluid of GA patients with different clinical characteristics

A comparison of the expression levels of PGC-1β, HIF-1α, and RETN in the synovial fluid of GA patients with different clinical stages, affected joints, disease courses, and annual attack frequencies revealed statistically significant differences (P<0.05), as shown in Table 3.

**Table 3:** Comparison of expression levels of PGC-1β, HIF-1α and RETN in synovial fluid of patients with different clinical characteristics in the GA group (x̄ ± s).

Clinical features	n	PGC-1β (pg/mL)			HIF-1α (ng/L)			RETN (ng/mL)		
		x̄±s	t/F	P	x̄±s	t/F	P	x̄±s	t/F	P
Clinical staging			4.037	0.020		71.207	0.001		47.009	<0.001
Acute stage	83	2.34±0.70			45.75±3.31			29.06±3.22		
Chronic phase	33	2.49±0.64			43.82±3.67			25.82±4.73		
Intermission period	18	2.83±0.59			35.50±2.43			20.38±2.25		
Affected joints<br>(number)			2.490	0.014		5.563	0.001		10.490	<0.001
≥5	54	1.92±0.55			45.21±2.78			29.04±1.71		
<5	80	2.18±0.62			42.63±2.53			25.62±1.94		
Course of the<br>disease (years)			3.104	0.002		4.242	0.001		6.713	0.001
≥5	53	1.88±0.54			44.99±2.85			29.12±1.93		
<5	81	2.20±0.61			42.72±3.14			26.51±2.36		
Annual frequency<br>of attacks (times)			4.496	<0.001		4.938	0.001		10.232	≤0.001
≥3	62	1.79±0.53			44.93±2.67			27.81±1.61		
≤3	72	2.26±0.66			42.59±2.79			24.75±1.82		

### Comparison of the expression levels of PGC-1β, HIF-1α and RETN with different degrees of joint destruction

The levels of PGC-1β in the serum and synovial fluid in the severe GA subgroup were lower than those in the mild GA subgroup, whereas the expression levels of HIF-1α and RETN were higher than those in the mild GA subgroup (P<0.05) - see Table 4.

**Table 4:** Comparison of expression levels of PGC-1β, HIF-1α and RETN with different degrees of joint destruction (x̄ ± s).

Group	n	PGC-1β (pg/mL)		HIF-1α (ng/L)		RETN (ng/mL)	
		Serum	Synovial fluid	Serum	Synovial fluid	Serum	Synovial fluid
Severe GA subgroup	78	1.76±0.43	1.82±0.45	48.14±8.51	53.78±8.68	27.69±5.22	30.06±5.47
Mild GA subgroup	56	2.38±0.35	2.43±0.38	37.16±7.93	42.55±8.13	22.78±4.16	25.19±4.72
t		8.880	8.248	7.577	7.583	5.832	5.377
P		≤0.001	≤0.001	≤0.001	≤0.001	≤0.001	≤0.001

### Comparison of the expression levels of bone destruction factors and inflammatory factors associated with different degrees of joint destruction

The expression levels of β-CTX and TRACP5b in the severe GA subgroup were greater than those in the mild GA subgroup (P<0.05), whereas the expression level of RANKL was lower than that in the mild GA subgroup (P<0.05) - see Table 5.

**Table 5:** Comparison of expression levels of bone destruction factors and inflammatory factors in different degrees of joint destruction (x̄ ± s).

Group	n	β-CTX (ng/mL)	RANKL (pg/mL)	TRACP5b (ng/mL)	TNF-α (pg/mL)	IL-1β (pg/mL)
Severe GA subgroup	78	1.46±0.45	121.53±50.16	3.97±1.16	29.93±5.31	125.31±23.52
Mild GA subgroup	56	1.08±0.32	179.70±81.95	2.82±0.90	18.96±3.42	84.52±14.43
t		5.411	5.085	6.197	13.564	11.509
P		<0.001	≤0.001	≤0.001	≤0.001	≤0.001

### Analysis of the relationships between serum PGC-1β, HIF-1α, and RETN and the degree of joint destruction, bone destruction factors, and inflammatory factors

There was a negative correlation between the level of joint damage and serum PGC-1β, β-CTX, TRACP5b, TNF-α, and IL-1β, and a positive correlation with RANKL. The degree of joint damage was positively correlated with HIF-1α, RETN, β-CTX, TRACP5b, TNF-α, and IL-1β, and negatively correlated with RANKL - see Table 6.

**Table 6:** Analysis of the relationship between serum PGC-1β, HIF-1α, RETN and the degree of joint destruction as well as bone destruction factors.

Indicator	PGC-1β (pg/mL)		HIF-1α (ng/L)		RETN (ng/mL)	
	r	P	r	P	r	P
Degree of joint<br>destruction	-0.562	≤0.05	0.579	≤0.05	0.613	≤0.05
β-CTX	-0.601	≤0.05	0.571	≤0.05	0.580	≤0.05
RANKL	0.524	≤0.05	-0.605	<0.05	-0.571	<0.05
TRACP5b	-0.612	<0.05	0.552	<0.05	0.609	<0.05
TNF-α	-0.614	<0.05	0.582	<0.05	0.581	<0.05
IL-1β	-0.682	≤0.05	0.607	<0.05	0.539	<0.05

### Analysis of the relationships between synovial fluid PGC-1β, HIF-1α, and RETN and the degree of joint destruction, bone destruction factors, and inflammatory factors

Synovial fluid PGC-1β, β-CTX, TRACP5b, TNF-α and IL-1β were negatively correlated with the degree of joint destruction and positively correlated with RANKL. There was a positive correlation between the degree of joint damage and HIF-1α and RETN, β-CTX, TRACP5b, TNF-α, and IL-1β, and a negative correlation with RANKL - see Table 7.

**Table 7:** Analysis of the relationship between synovial fluid PGC-1β, HIF-1α, RETN and the degree of joint destruction, bone destruction factors.

Indicator	PGC-1β		HIF-1α		RETN	
	r	P	r	P	r	P
Degree of joint<br>destruction	-0.568	≤0.05	0.594	≤0.05	0.617	≤0.05
β-CTX	-0.614	≤0.05	0.576	≤0.05	0.583	≤0.05
RANKL	0.527	≤0.05	-0.611	≤0.05	-0.575	≤0.05
TRACP5b	-0.622	≤0.05	0.572	<0.05	0.616	<0.05
TNF-α	-0.643	≤0.05	0.594	≤0.05	0.582	<0.05
IL-1β	-0.691	≤0.05	0.614	≤0.05	0.559	<0.05

## Discussion

HIF-1α transcriptional activity can regulate the compensatory response to tissue hypoxia [10]. Previous reports have shown that GA is associated with hypoxia in the local microenvironment [11][12]. Research [13] has indicated that elevated expression of serum HIF-1α is a risk factor for the onset of primary GA and is abnormally increased in patients with primary GA. The results of this investigation demonstrate that the GA group's serum HIF-1α expression level was higher than the control group's. There are differences in the expression of serum HIF-1α among GA patients with different clinical stages, affected joints, disease courses and annual attack frequencies. Moreover, the correlation analysis revealed a negative correlation between RANKL and the degree of joint degeneration, as well as a positive correlation with HIF-1α, β-CTX, and TRACP5b. An elevated level of serum HIF-1α may aggravate the inflammatory response and joint destruction in patients with GA. The possible reasons are as follows: During hypoxia, the degradation of HIF-1α is blocked, allowing it to accumulate and enter the cell nucleus, which promotes the transcription of hypoxia response genes in the local joint tissues of GA patients and thereby exacerbates the degree of hypoxia [14][15]. On the one hand, HIF-1α is related to the hypoxia response. On the other hand, its expression level is regulated by inflammatory factors, which gradually increase with increasing inflammation and intensify the hypoxia response in joint tissues, creating a vicious cycle [16][17][18]. TNF-α and IL-1β showed favourable correlations with HIF-1α, indicating that HIF-1α is closely related to inflammatory mediators and can serve as an indicator for the clinical assessment of GA conditions, providing a potential basis for clinical diagnosis and treatment.

Studies have shown that PGC-1β and HIF-1α are positively regulated by the c-Myc gene, and that a high level of HIF-1α can indirectly reduce PGC-1β expression [19]. Research has shown that PGC-1β is abnormally expressed in the synovium of patients with rheumatoid arthritis [20]. This study revealed that the expression level of serum PGC-1β in the GA group was abnormally decreased, and in GA patients with varying clinical stages, affected joints, disease courses, and annual attack frequencies, HIF-1α and RETN in serum and synovial fluid exhibit opposite states, suggesting that PGC-1β may also be involved in the progression of GA. Furthermore, it inhibits mitochondrial oxidative phosphorylation, indicating a negative regulatory effect between PGC-1β and HIF-1α. HIF-1α may inhibit mitochondrial biogenesis by negatively regulating PGC-1β [21]. In this study, the expression levels of PGC-1β in the serum and synovial fluid of the severe GA subgroup were lower than those in the mild GA subgroup. Further correlation analysis revealed that PGC-1β in the serum and synovial fluid was negatively correlated with the degree of joint destruction, P-CTX, TRACP5b, TNF-α, and IL-1β and positively correlated with RANKL, suggesting that PGC-1β is associated with GA, which is closely related to the degree of joint damage and inflammation. This may be related to the cytokine signal transduction effect of PGC-1β, which, as a coactivating transcription factor, may be involved in the inflammatory regulation of GA.

RETN is an endocrine hormone that is specifically produced in adipocytes, is rich in cysteine, and is usually expressed in immune cells, spleen cells, and pancreatic islet cells [22][23]. One study [24] suggested that RETN is associated with inflammation, obesity, and stress response mechanisms, and has a potential clinical diagnostic role. Another study [8] revealed that serum RETN in GA patients was positively correlated with pain and bone destruction. RETN, an adipocytokine, regulates energy metabolism, fat content, body weight and insulin resistance [25][26][27][28]. The expression levels of RETN in the serum and synovial fluid of GA patients in the acute stage are higher than those in the chronic and intermittent stages. The expression level of RETN in GA patients with ≥5 affected joints, a disease course ≥5 years, and an annual attack frequency ≥3 times was greater than that in GA patients with <5 affected joints, a disease course <5 years, and a yearly attack frequency <3 times. Moreover, relevant studies have demonstrated that RETN can mediate the secretion of inflammatory mediators, thereby promoting the progression of joint inflammation and indirectly contributing to joint bone destruction [29][30][31][32]. Therefore, this study attempted to analyse the association between serum RETN and GA joint destruction. RETN showed a negative correlation with RANKL and a positive correlation with the degree of joint degradation, as well as with β-CTX, TRACP5b, TNF-α, and IL-1β. This may be due to the elevated blood uric acid content in GA patients, uric acid deposition at the joints, increased blood circulation and local RETN of the joints, which promote macrophage infiltration and exacerbate GA joint injury [33][34][35].

In conclusion, significant abnormalities are observed in PGC-1β, HIF-1α, and RETN in GA patients. Moreover, pGC-1β, HIF-1α, and RETN are closely related to the degree of joint destruction, bone destruction factors, and inflammatory levels in GA. Combined detection can offer a novel approach to the clinical assessment of GA severity.

## Dodatak

### Conflict of interest statement

All the authors declare that they have no conflict of interest in this work.
